# Optimizing thermocouple’s ZT through design innovation

**DOI:** 10.1038/s41598-021-98562-z

**Published:** 2021-09-29

**Authors:** Tinggang Zhang

**Affiliations:** Formerly, University of Alaska Fairbanks, 306 Tanana Drive, Duckering Building, Fairbanks, AK 99775 USA

**Keywords:** Devices for energy harvesting, Mechanical engineering

## Abstract

This work demonstrates that in parallel with the one existed at high doping concentration, there also exists an optimal combination of the transport properties of a thermoelectric material at low doping concentration as the curve of the relation between electrical conductivity and doping concentration is rigidly shifted toward that direction without disturbing the Seebeck coefficient and the thermal conductivity. Based on this finding, a new thermocouple design that uses low doping legs and high doping semiconductors as the external carrier injectors surrounding the legs is developed. The analytical model developed for the new thermocouple indicated that its efficiency and power output could be more than tripled as compared to those of the original design. A single thermocouple made of Silicon semiconductors was simulated numerically using different sets of input parameters. The results showed that the density of the externally injected carriers played a significant role in enhancing the thermocouple’s efficiency and power output.

## Introduction

**Figure 1:**
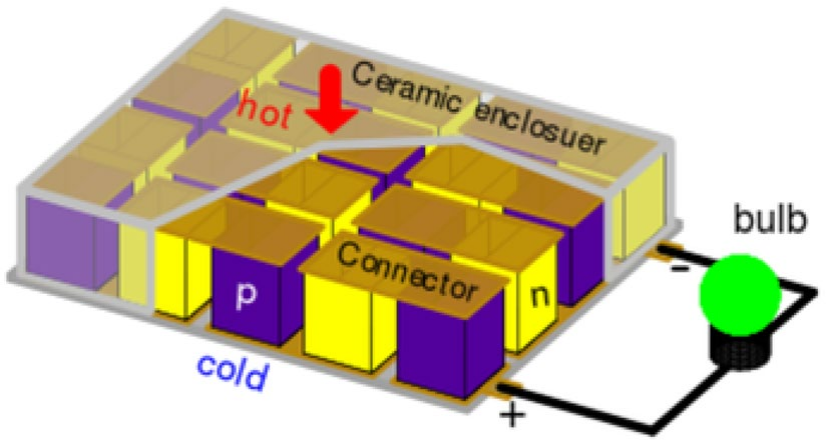
A typical flat planar thermoelectric generator. It is usually assembled from a numbers of thermocouples made of a *p*- and an *n*-type semiconductor legs. These legs are connected through interconnectors (metal strips in brown) and sandwiched between a hot and a cold electrical insulators (ceramics in gray) so that the electric current flows in series and the heat current flows in parallel through these legs. These modules are sandwiched between heat exchangers (not shown) that supply heat to the hot side and remove the exit heat from the cold side to maintain a temperature difference between the two sides. The external terminals of these modules are connected to an electrical load (bulb) to form a closed system for electricity generation.

Enhancing energy-use efficiency through thermoelectric generator, a solid-state device that can reliably convert heat released from vehicle exhaust and various manufacturing processes directly into electricity without the need for any moving mechanical components, constitutes one of the important measures toward building a sustainable environment. Such a technique can not only reduce fuel consumption that is depleting the limited oil reserves but also lower greenhouse gas emissions that have a tremendous detrimental impact on our environment. More than half of the energy supplied to the current thermal-process-based power generation systems or combustion engines is lost mostly as heat^1,2^. The potential to convert the released heat into electricity through thermoelectic generator is significant. Figure 1 shows the architecture of a flat planar thermoelectric power generator used in most of the contemporary designs. Evidently, the power output and the efficiency of a generator is determined by the atomic structure of the semiconductor legs, the design of the leg, thermocouple or module, the heat exchangers, and the circuit of the electricity harnessing unit. In the current generator design, the atomic structure of a semiconductor leg plays a limiting role in determining the generator’s efficiency. The quality of a semiconductor as a thermoelectric leg is measured by a dimensionless parameter called “figure of merit” defined as ${zT}=\alpha ^2\,\sigma \,T/\kappa$, where $\alpha$ is the Seebeck coefficient, $\sigma$ the electrical conductivity, and $\kappa$ the thermal conductivity. For a thermocouple or a module, it is transformed into ${{ZT}}=\alpha ^2\,T/(R\,K)$, where *R* is the electrical resistance and *K* the thermal conductance, to take into account of the effects of its geometry and size.

For a thermoelectric leg with temperature-independent transport properties, its efficiency under a given temperature difference can be expressed as a function of its *zT* by:

1 $$\begin{aligned} \eta = \frac{(T_h-T_c)}{T_h}\left[ \frac{\sqrt{1+zT_m}-1}{\sqrt{1+zT_m}+(T_c/T_h)} \right] \end{aligned}$$

where ${T_h}$ and ${T_c}$ are the absolute temperatures at the hot- and the cold-ends of the leg, respectively, and $T_m$ is the mean temperature measured between the two ends. Clearly, a large *zT* of a thermoelectric leg is crucial for the efficiency of a generator and is the ultimate goal in the search for a good thermoelectric material besides its abundance and cost. “Untuned” bulk thermoelectric materials have the *zT*s much less than 1.0 and can hardly be useful for high-efficient power generation. These materials have to be “tuned” to have (1) large power factor $\alpha ^2\,\sigma$ and (2) lower thermal conductivity $\kappa$ to attain large *zT*s. However, it has proved to be a challenging task to meet both of these two requirements in a material since its transport properties are intrinsically related and are functions of the band structures, doping concentrations, and scattering processes among others. As it will be showed in later section that $\alpha$ and $\sigma$ generally vary in a reciprocal manner, which makes any improvement on *zT* difficult.

**Figure 2:**
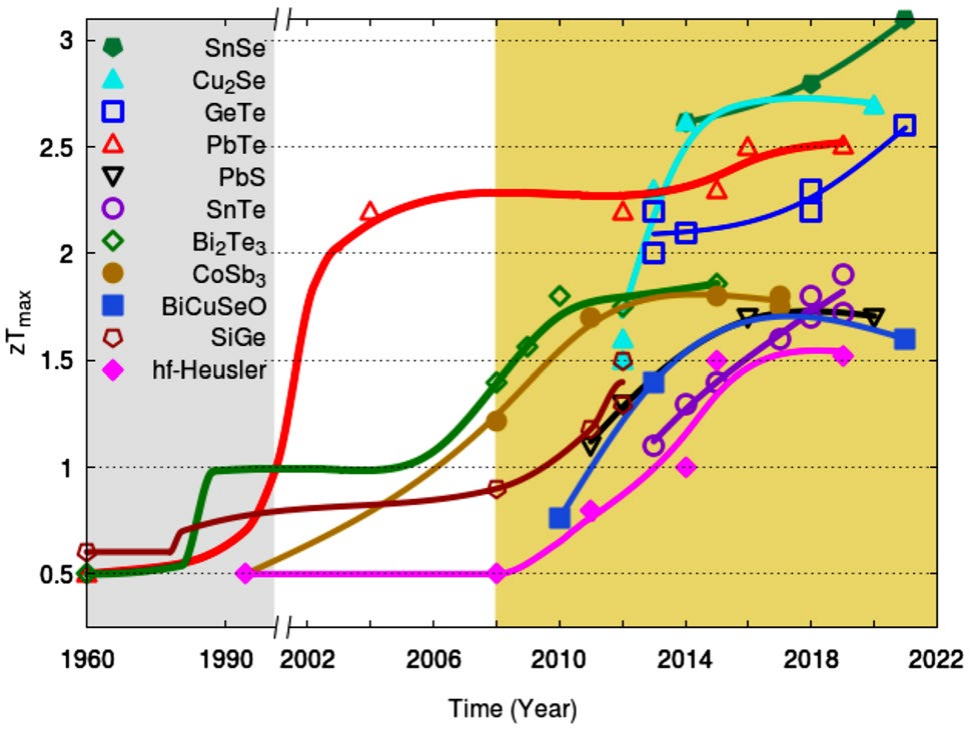
Upper *zT*-envelopes constructed from the existing data for: SnSe^9–11^, Cu2Se^12–16^, GeTe^17–22^, PbTe^23–27^, PbS^28–31^, SnTe^32–39^, Bi$_2$Te$_3$^40–44^, Skutterudites^45–49^, BiCuSeO^50–52^, SiGe^53–56^, Half-Heuslers^57–61^. The lines are only for differentiating one group of materials from the other.

Clearly, an ideal thermoelectric material should have a large Seebeck coefficient and behave like a crystalline solid for its charge carrier transport and like an amorphous solid for its phonon transport, so called “phonon glass/electron crystal, PGEC^3,4^. Through many decades’ search, a deep insight of the intrinsic nature of a good thermoelectric material has been gained, theories, various synthesis technologies, and treatment techniques have been developed toward realizing the ideal thermoelectric material. These include low-dimensional theory^5,6^, band engineering, structural modification, phase manipulation, etc. (for a comprehensive review in this regard, refer to Ref^7^). These efforts have led to the developments of many new materials and the achievement of *zT* as high as 3.1. These advances in the quality of thermoelectric materials represented by the improvement made on *zT*s have been compiled into historical charts for typical materials^7,8^ . Based on these charts, the upper envelopes of the *zT*s of several classes of thermoelectric materials can be drawn as shown in Fig. 2.

Translating high *zT* thermoelectric materials into a high-efficient generator has proven to be challenging not only because the transport properties of both the *n*- and the *p*-type semiconductor legs are temperature-dependent but also because their optimum *zT*s are different both in the magnitude and at the temperature level and are only attainable within a small fraction of the operating temperature range of the device. Furthermore, construction of a generator needs other components such as the interconnectors, thermal insulating ceramics, heat exchangers, and electricity extracting unit. These components introduce additional electrical and thermal resistance that have to be minimized to make the device as efficient as its legs. Consequently, module design plays an important role in realizing the efficiency of thermoelectric legs at the generator level and currently lags far behind the advances of thermoelectric materials.

Design optimizations so far are limited to the parameters including leg length, cross-sectional area, and shape; module fill factor and the electrical and thermal resistance induced by the interfaces between the leg and the interconnector and between the interconnector and the thermal insulator. It was found that an optimal *ZT* could be obtained when the relation ${A_p}{L_n}/({A_n} {L_p}) = \sqrt{{{\kappa }_n}{{\rho }_p}/({{\kappa }_p}{{\rho }_n})}$ for the two legs was satisfied^62^, where *A* and *L* are, respectively, the cross-sectional area and length of the leg and the subscripts *n* and *p* are leg types. Numerical simulation showed that this analytical relation was approximately true even when the temperature-dependent transport properties of the materials and the electrical and thermal contact resistances were taken into account^63^. The numerical simulation further showed that a similar power output per unit module area obtained with a greater number of longer legs could be realized with a smaller number of shorter legs when the thermal contact resistance was neglected.

Early investigation^64^ indicated that different shapes of a constant cross-sectional area along a leg had little effect on power generation and efficiency of the module. However, a leg with variable cross-sectional area showed improved power generation and efficiency^65,66^. Among different leg shapes investigated, pyramidal leg^66,67^ showed a higher output power density than cuboid, exponential^68,69^, and quadratic shaped legs^70^. Subsequently, a laboratory-scale thermoelectric module with pyramid-shaped leg was recently built for proof-of-concept^71^. The experimental data confirmed the analytical prediction. A pyramid-shaped leg can increase the temperature gradient along the leg, thus the Seebeck voltage, by lowering its thermal conductance. Furthermore, it can take into account of the Thomson effect which is usually ignored in the leg with a constant cross-sectional area. The laboratory-scale module showed a 67% increase in power generation compared with the module having cuboid legs. More leg shapes , including cuboid, trapezoid, hourglass, Y-shaped, and hollow legs and their different arrangements were modeled using the finite element method^72^. The numerical results showed that the hourglass shaped leg had more than doubled electrical potential and the maximum power output compared to the cuboid leg when a constant temperature is maintained at the hot end and a natural convection is applied at the cold end.

Three approaches, i.e., functionally graded thermoelectric material (FGTEM), segmented thermoelectric legs, and cascaded thermoelectric modules, are commonly used to improve the low average *zT* within a large operating temperature range. The fundamental concept of FGTEM is to synthesize a leg material to have an optimum *zT* all the way from the hot-end to the cold-end through locally selecting a particular material, microstructure, or composition. Several techniques, including tuning the amount of dopant at different spatial positions of^73^ and creating a carrier concentration or a composition gradient in a material^73–82^, have been proposed to synthesize FGTEMs. FGTEM has the advantages to have a gradual phase transition without inducing any interface resistances. However, the synthesizing technology so far is challenging. A segmented FGTEM that joins the existing thermoelectric materials to fit the *zT* profile of a thermoelectric leg with their optimum *zT*s was proposed by Ioffe in his patent application^83^ and gains increased interest since^83–87^. Several promising designs^88,89^ have been studied and showed improved efficiencies. A shortcoming of the segmented legs is the presence of the large interface resistances at the junctions between consecutive sections. Furthermore, the materials of different segments have to be compatible to make a high-efficient leg^90,91^. Cascade modules can avoid the electrical contact resistance problem by stacking multiple stages of thermocouples with different materials and using separated electric circuit for each stage to improve its performance within large temperature difference. Cascade module is complicated to implement.

Clearly, current device design optimizations can only produce an optimal efficiency within the range limited by its leg’s *zT* which is yet below the anticipated level, $zT>3$ which is needed to make the device potentially competitive with the traditional thermal-process-based energy conversion technology. In this work, a new design approach is introduced to develop a thermoelectric generator with conversion efficiency and power output that can potentially exceed those of the legs’ materials. The existence of an optimal combination of the transport properties of a thermoelectric material at low doping concentration under an ideal condition is firstly illustrated. A new thermocouple design that uses low doping legs and high doping semiconductors as external carrier injectors surrounding the legs is then introduced. Finally, an example is provided to demonstrate the potential of the new approach in enhancing the device efficiency.

## Results

### Optimal figure of merit

**Figure 3:**
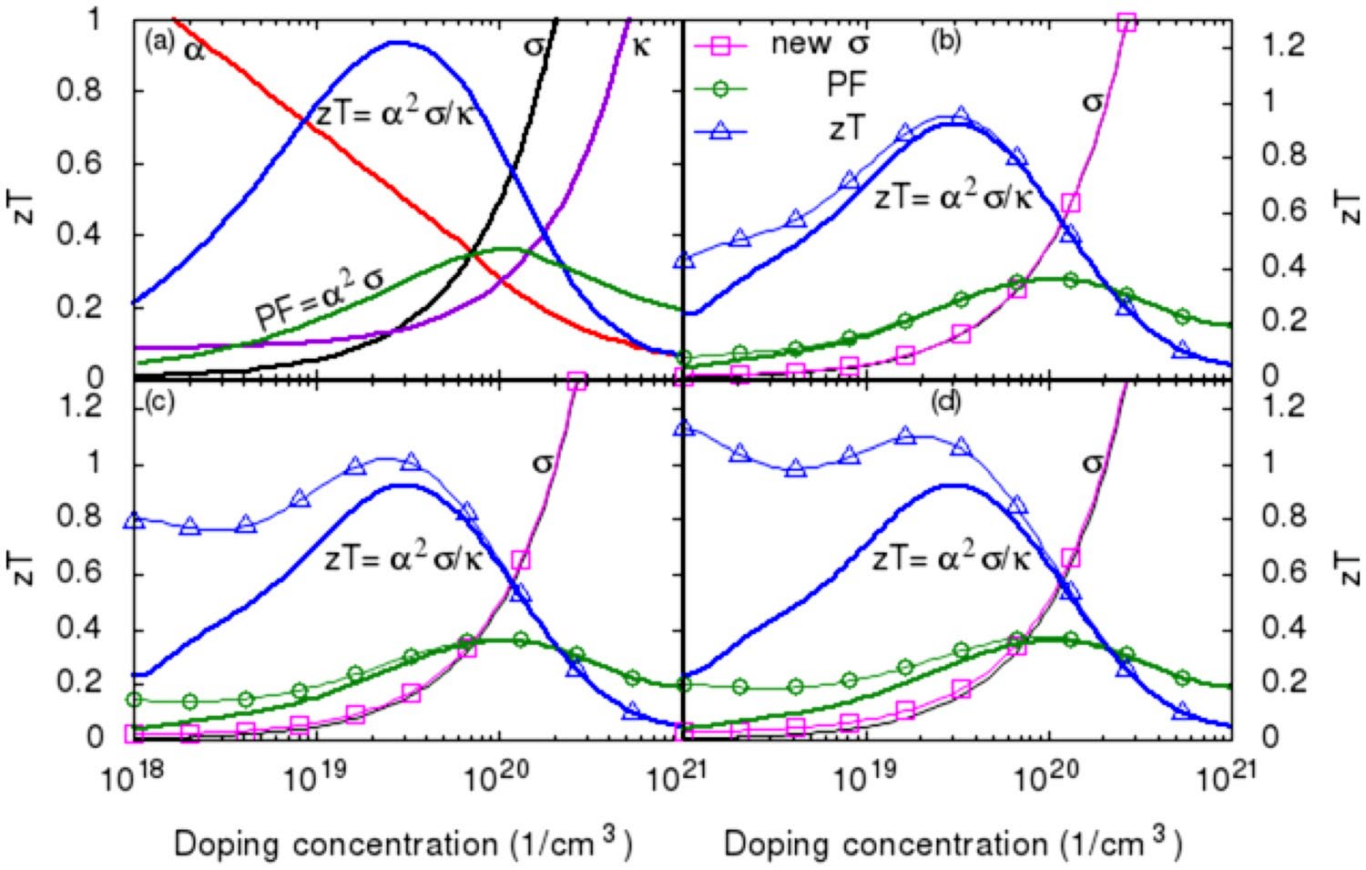
Optimizing *zT* through tuning carrier concentration. (**a**). Thermoelectric transport properties as functions of carrier concentration. Reproduced from Ref.^93^. (**b**). Shifting the $\sigma$-curve by 10$^{18}$ cm$^{-3}$ toward lower doping concentration without disturbing the $\alpha$- and $\kappa$-curves leading to a 83% increase in the electrical conductivity, electric current, power factor, and *zT*, and a 68% increase in the conversion efficiency at $N_D=10^{18}$. (**c**). Shifting the $\sigma$-curve by $3\cdot 10^{18}$ cm$^{-3}$ leading to a more than twofold increase in the electrical conductivity, electric current, power factor, and *zT*, and a more than one and half fold increase in the conversion efficiency. (**d**). Shifting the $\sigma$-curve by $5\cdot 10^{18}$ cm$^{-3}$ leading to a more than three and half fold increase in the electrical conductivity, electric current, power factor, and *zT* and nearly two and half increase in the conversion efficiency. It can be noticed that the optimum *zT* also shifted toward low doping concentration.

In addition to depicting the complex nature of the intrinsically dependent transport properties as stated in the Introduction, Fig. 3a also shows clearly that lower doping concentration yields two best transport properties, i.e., Seebeck coefficient and thermal conductivity, while higher doping concentration results in only one best transport property, electrical conductivity, for thermoelectric applications. Such a pattern is also observed in other materials^92^. In this regard, using low doping concentration as a starting material to optimize its thermal-to-electrical conversion efficiency could have certain advantages over the high-doping concentration approach. However, low doping material has limited charge carriers to work with to improve its electrical conductivity. This could be a major barrier in searching for an ideal thermoelectric material with low doping concentration. In this section, it will be shown that an optimal combination of the transport properties also exists at lower doping concentration for certain materials with their transport properties have the similar trends as those shown in Fig. 3a.

Assuming the electrical conductivity curve shown in Fig. 3a can be rigidly shifted toward lower doping concentration without disturbing the Seebeck coefficient and the thermal conductivity curves, a new set of transport properties can be obtained as shown in Fig. 3. Figure 3b to d are the results obtained by shifting the $\sigma$-curve by $10^{18}$, $3\cdot 10^{18}$, and $5\cdot 10^{18}$ cm$^{-3}$, respectively, toward low doping concentration. For a thermoelectric leg with $A_n$=0.267 cm$^2$, $L_n$=2 mm, and initial doping concentration $N_D=10^{18}$ cm$^{-3}$, the improvemed electrical conductivity, power factor, and *zT* are extracted from those shifted curves shown in Fig. 3 and are presented in Table 1. The intrinsic electrical resistance is estimated by ${R_I}=L_n/\sigma /A_n$. The load resistance is estimated by ${R_L}=10^2/\sigma$ to approximately match the intrinsic electrical resistance. The electric current is then calculated by ${I}=\alpha \,{\Delta T}/(R_L+R_I)$. The conversion efficiency is determined by Eq. (1). The electrical conductivity increased by about 83% for the case of ${\Delta n}=10^{18}$, by about 238% for $\Delta n=3\cdot 10^{18}$, and by about 378% for $\Delta n=5\cdot 10^{18}$, respectively. Similar increase scales are also found for electric current, power factor, and figure of merit. The conversion efficiency increased by about 68% from 0.026 to 0.044 in the first case, by about 169% from 0.026 to 0.07 in the second case, and by about 242% from 0.026 to 0.089 in the third case. It can be noticed from Fig. 3d that the optimum *zT* is also shifted toward low doping concentration, which indicates that the optimum *zT* can also be attainable in low doping materials through tuning the electrical conductivity.

**Table 1:** The electrical conductivity $\sigma$, electric current *I*, power factor *PF*, figure of merit *zT*, and the conversion efficiency $\eta$ vary with the incremental doping concentration, $\Delta n$.

$\Delta n$	$\sigma$	$R_L$	$R_I$	*I*	PF	*zT*	$\eta$
($10^{18}\,$cm$^{-3}$)	(S/cm)	$(\Omega )$	$(\Omega )$	(A)			
0	3160	0.032	0.023	1.72	0.043	0.236	0.026
1	5782	0.017	0.013	3.14	0.078	0.431	0.0437
3	10686	0.0094	0.007	5.81	0.145	0.797	0.07
5	15113	0.0066	0.005	8.22	0.205	1.127	0.089

### New thermocouple design

Shifting $\sigma$-curve without disturbing $\alpha$- and $\kappa$-curves implies that the charge carriers are injected into the leg without altering the lattice structure of the material. Consequently, the Seebeck coefficient and the thermal conductivity remain the same as those before shifting the $\sigma$-curve. Applications of such a concept can be found in most of the semiconductor devices, typically in bipolar junction transistors (BJTs) and metal-oxide-semiconductor field effect transistors (MOSFETs). In BJT, nearly 100% of the charge carriers of emitter is injected into the collector through the two p-n junctions to increase the collector’s electric current without altering its lattice structure. In MOSFET, a significant amount of carriers of the *n*- or *p*-channel is injected into the drain to increase its electric current without altering its lattice structure. Injecting charge carriers into a thermoelectric leg without disturbing its lattice structure is one of the aspects on which the new design concept is based. The another aspect is to use lower doping concentration as the starting material that has both the best Seebeck coefficient and the best thermal conductivity, leaving the electrical conductivity to be improved by injecting carriers from the leg’s surrounding boundary using higher doping concentration materials. The theory behind this concept can be reasoned by the following three processes.

First, when a semiconductor with high carrier concentration is joined with a leg that has lower carrier concentration, the concentration gradient at the interface causes the carriers to diffuse from the higher concentration material to the lower concentration leg. As a result, the carrier concentration near the boundary of the leg increases, which leads to higher electric current density. From Drude’s model, $\sigma ={q}{n_e}{\mu }$, where *q* is electric charge, $n_e$ carrier concentration, and $\mu$ carrier mobility, it is known that the electrical conductivity increases as the carrier concentration increases. Since the higher carrier concentration only exists near the leg boundary, the interaction between these injected carriers and the carriers and phonons of the leg is supposed to be minimal. It can be reasonably anticipated that the injected carriers cause little disturbance to the leg’s lattice structure, thus the Seebeck coefficient and the thermal conductivity remain unchanged. Consequently, the figure of merit of the leg increases with the increase of injected carrier concentration.

Second, as carriers continue to move across the interface into the leg, some uncompensated positive ions remain near the interface on the side of the material with high carrier concentration. An electric field with a direction from the positive charge toward the negative charge is build-up at the interface. This electric field creates a barrier potential preventing further carrier diffusion until an equilibrium state is reached. This electric field pulls the injected carriers toward leg’s exterior boundary. As a result, the carrier concentration at the boundary is significant higher than that of the leg, which can prevents electrons of a *n*-type leg move toward the boundary where, otherwise, an infinitive carrier recombination rate exists. Consequently, this carrier concentration gradient reduces the carrier recombination rate at the boundary such that electron mobility near the boundary is improved. From Drude’s model, it is also known that the electrical conductivity increases with electron mobility.

Third, as the higher carrier concentration material is joined with the leg at the leg’s exterior boundary, the originally free boundary of the leg becomes constrained. Thus, its phonon waves also become constrained at the boundary. Consequently, the higher carrier concentration material can be an additional freedom to modulate the lattice vibration mode of the leg to further lower its lattice thermal conductivity.

**Figure 4:**
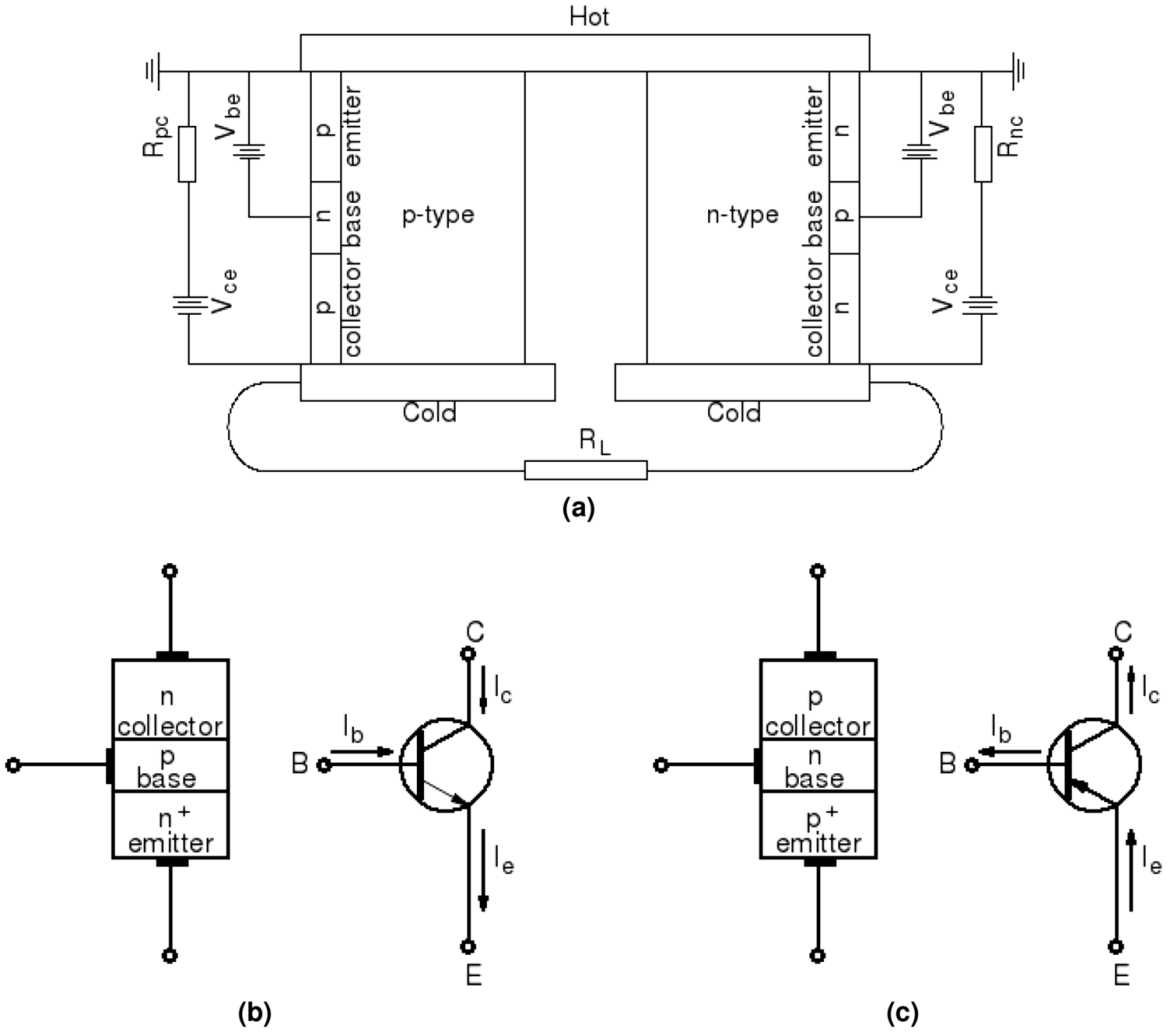
(**a**) Schematics of the new thermocouple design including a *p*-type and an *n*-type thermoelectric legs, conductor strips at the hot end and the cold ends and a electrical load resistance, $R_L$. An *n*-*p*-*n* bipolar junction resistor (not to scale) is joined with the *n*-type leg at the exterior surface and a *p*-*n*-*p* BJT is joined with the *p*-type leg. These two BJTs are common-emitter configured. ${V_{be}}$ is the bias between the base and the emitter. ${V_{ce}}$ is the bias between the collector and the emitter. ${R_{nc}}$ and ${R_{pc}}$ are the load resistance for the *n*-*p*-*n* and *p*-*n*-*p* transistors, respectively. (**b**) Symbols of an *n*-*p*-*n* transistor. (**c**) Symbols of a *p*-*n*-*p* transistor.

To investigate the effects of the injected carriers on the transport properties and on the efficiency of a thermocouple, a common-emitter bipolar junction transistor is used in this work to substitute the high-carrier-concentration semiconductor. The BJTs are joined or embedded on the exterior boundaries of both the *n*- and the *p*-type legs. Figure 4 shows a schematics of the design of the new thermocouple. By adjusting the base current of the transistor, the amount of the injected carriers into the thermoelectric legs can be modulated to obtain an optimal injected carrier concentration that maximizes the efficiency and power output of the thermocouple.

#### Electric characteristics of BJT

Models to determine the current-voltage characteristics of an *n*-*p*-*n* bipolar transistor is briefly introduced in this section (see Methods for details and variable definitions). A bipolar junction transistor is made of three regions and two *p*-*n* junctions as shown in Fig. 4b for an *n*-*p*-*n* transistor and 4c for a *p*-*n*-*p* transistor. These three regions are designated as: emitter, base, and collector with the base sandwiched between the emitter and the collector. The emitter and the base regions form the first *p*-*n* junction. The second *p*-*n* junction is formed between the base and the collector regions. The emitter has the highest doping, the collector has the lowest doping. The doping concentration in the base is smaller than that in the emitter but higher than that in the collector. Three terminals connect, respectively, to each region. Based on the bias applied at these terminals, a BJT can be operated in four different modes: forward active, saturation, cutoff, and inverted. For each operating mode, the bias sets the level of barrier potentials at the two metallurgical junctions to modulate the carrier flow from one region to another.

In the new thermocouple design, the normal operating condition, i.e., the forward active mode is used, in which the emitter-base junction is forward biased, whereas the collector-based junction is reverse biased. Based on ideal *p*-*n* junction theory, the emitter, base, and collector currents, ${I_e}$, ${I_b}$, and ${I_c}$, respectively, can be expressed in the following compact form^93–96^

2a $$\begin{aligned} I_e&= I_{ne} + I_{pe} = a_{11}\,\left( e^{{\eta _{be}}} -1\right) + a_{12}\,\left( e^{{\eta _{bc}}} -1\right) \end{aligned}$$

2b $$\begin{aligned} I_c&= I_{nc} + I_{pc} = a_{21}\,\left( e^{\eta _{be}} -1\right) + a_{22}\,\left( e^{\eta _{bc}} -1\right) \end{aligned}$$

2c $$\begin{aligned} I_e&= I_b + I_c \end{aligned}$$

where

2d $$\begin{aligned}&a_{11} = q\,S\,\left[ \frac{D_{pe}\,p_{e0}}{L_{pe}}\, \coth {\left( \frac{x_e}{L_{pe}}\right) } + \frac{D_{nb}\,n_{b0}}{L_{nb}}\, \coth {\left( \frac{W}{L_{nb}}\right) }\right] \end{aligned}$$

2e $$\begin{aligned}&a_{12} = -\frac{q\,S\,D_{nb}\,n_{b0}}{L_{pe} \sinh {\left( \frac{W}{L_{nb}}\right) }} a_{21}=-a_{12} \end{aligned}$$

2f $$\begin{aligned}&a_{22} = -q\,S\,\left[ \frac{D_{nb}\,n_{b0}}{L_{nb}}\, \coth {\left( \frac{W}{L_{nb}}\right) } + \frac{D_{pc}\,p_{c0}}{L_{pc}}\, \coth {\left( \frac{x_c}{L_{pc}}\right) }\right] , \end{aligned}$$

When $I_b$ and $V_{bc}$ are given, the common-emitter collector current can be obtained from Eq. (2) and expressed as:

3 $$\begin{aligned} I_c = \frac{a_{21}}{a_{11}-a_{21}}\,I_b + \left[ \frac{a_{11}\,a_{22} -a_{12}\,a_{21}}{a_{11}-a_{21}}\right] \left( e^{V_{bc}/V_{th}} -1\right) \end{aligned}$$

The current gain of a common-base bipolar junction transistor, denoted as ${\alpha _F}$, is defined as the ratio of collector current to emitter current, i.e.:

4 $$\begin{aligned} \alpha _F = \frac{\partial I_{nc}}{\partial I_e}\,\quad \,\Rightarrow \, \quad \,\alpha _F = \frac{\partial I_{nc}}{\partial I_{ne}}\, \frac{\partial I_{ne}}{\partial I_e}\,\quad \,\Rightarrow \,\quad \, \alpha _F = \alpha _T\,\gamma \end{aligned}$$

where ${\alpha _T}$ is defined as the base transport factor and ${\gamma }$ is the emitter injection efficiency. These current gain factors can be evaluated by:

5 $$\begin{aligned} \alpha _T \approx \frac{1}{\cosh (W/L_{nb})} \approx 1\,-\,\frac{1}{2} \left( \frac{W}{L_{nb}}\right) ^2&\quad \quad {\text {and}} \quad \quad \gamma = \frac{D_{nb}\,N_{De}\,x_e/(D_p\,N_{Ab}\,W)}{1 + \left[ D_{nb}\, N_{De}\,x_e/(D_p\,N_{Ab}W)\right] }. \end{aligned}$$

The common-emitter current gain, ${\beta }$, is defined as the ratio of collector current to base current, i.e., $\beta = {\partial I_c}/{\partial I_b}$. Using Eq. (2c), it can be expressed in the common-base current gain as:

$$\begin{aligned} \beta = \frac{\partial I_c}{\partial I_b} = \frac{\partial I_c}{\partial (I_e-I_c)} = \frac{\partial I_c/\partial I_e}{1 - \partial I_c/\partial I_e} = \frac{\alpha _F}{1 - \alpha _F}. \end{aligned}$$

In most modern bipolar junction transistors, $\alpha _F \approx 1$, hence $\beta$ is significantly larger than 1.

**Figure 5:**
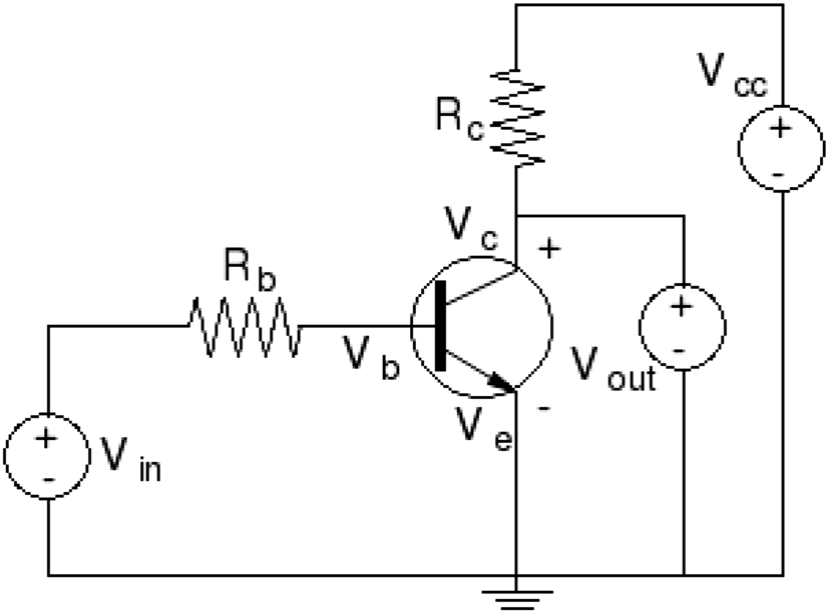
A schematics of a simple amplifier. It is composed of a common-emitter n-p-n bipolar transistor and two resistors. ${V_{cc}}$ is the power supply hold at a constant positive voltage. ${V_{in}}$ is the input signal. $V_{out}=V_{ce}$ is the output signal. The $V_{cc}$, ${R_c}$, collector, and emitter form an output loop. The ${V_{in}}$, ${R_b}$, base, and emitter form an input loop.

#### Amplification with bipolar transistors

Voltages and currents can be amplified by a bipolar transistor in combination with other components. This amplification functionality of a bipolar transistor is used here to modulate the amount of charge carriers injected into the thermoelectric legs in the new thermocouple design. Figure 5 shows a schematics of a simple amplifier isolated from Fig. 4. The amplifier consists of an *n*-*p*-*n* bipolar transistor in a common-emitter configuration and two resistors to modulate the voltages at the base and the collector. $V_{cc}$ is the supplied positive voltage hold at a constant value. The input signal is delivered by the source voltage, $V_{in}$. The output signal, $V_{out}$ which is equal to $V_{ce}$, is measured between collector and emitter terminals. Using Ohm’s and Kirchhoff’s laws, the relationship between the output voltage and the input voltage can be determined, respectively, from the collector-emitter loop and the base-emitter loop as:

6 $$\begin{aligned} V_{in} = R_b\,I_b + V_{be}\,\quad \,{\text { and }}\,\quad \, V_{cc} = R_c\,I_c + V_{ce} \end{aligned}$$

For a common-emitter transistor in forward active mode, $I_c=\beta \,I_b$ and $V_{be}$ is a constant. The amplified voltage, i.e., $V_{out}$ can be expressed as^97^:

7 $$\begin{aligned} V_{out} = V_{ce} = V_{cc}\,- \beta \,\frac{R_c}{R_b}\,\left( V_{in} - V_{be}\right) \end{aligned}$$

Since $V_{be}$ is a constant, any change in the input signal $V_{in}$ leads to a change in the output signal ${V_{out}}$. The variation of the output voltage is proportional to the change of the input voltage by an amplification factor expressed as:

8 $$\begin{aligned} {\Delta V_{out}} = - \beta \,\frac{R_c}{R_b}\,{\Delta V_{in}} \end{aligned}$$

Multiplying the second equation of Eq. (6) by the collector current, the supplied power distribution between the resistor $R_c$ and the transistor is obtained as:

9 $$\begin{aligned} I_c\,V_{cc} = R_c\,I_c^2\,+ I_c\, V_{ce} \end{aligned}$$

where $I_c\,V_{cc}$ is the supplied power, $R_c\,I_c^2$ is the power dissipated in the resistor, and $I_c\,V_{ce}$ is the power used by the transistor to amplify the input signal. The later term is the power used in the new thermocouple design to improve the leg’s electrical conductivity and the terminal voltage.

#### Thermocouple power gain

From heat and electric current coupled equations, heat balance equations can be reduced for a thermoelectric leg. The heat balance equations at the hot and the cold ends of the leg are, respectively, expressed as^98,99^:

10a $$\begin{aligned}&{Q_H} = \alpha \,T_h\,{I_{TEG}}\,- \frac{1}{2}\,R\,I_{TEG}^2\, + {K_{TEG}}\, \Delta T \end{aligned}$$

10b $$\begin{aligned}&{Q_C} = \alpha \,T_c\,I_{TEG}\,+ \frac{1}{2}\,R\,I_{TEG}^2\, + K_{TEG}\, \Delta T \end{aligned}$$

The voltage of the leg between the hot and the cold terminals is given by $V_{TEG}=\alpha \,\Delta T$, where $\Delta T=T_h-T_c$. The electric current flowing through the leg is calculated by Ohm’s law as $I_{TEG}=V_{TEG}/(R_I+R_L)$, where $R_I$ and $R_L$ are, respectively, the intrinsic and load resistances. The output power of the leg is:

11 $$\begin{aligned} {P_{0}} = I_{TEG}\,V_{TEG}= I_{TEG}^2\,R_L. \end{aligned}$$

For the leg of the new thermocouple, there exist two electric currents flowing through it. One is from the thermocouple loop and the other is from the bipolar transistor loop as shown in Figs 4 and 5. As it is pointed out earlier that the power $I_c\,V_{ce}$ used by the transistor to amplify the input signal can improve both the electrical conductivity and the terminal voltage in the new thermocouple design. As a first-order approximation, the current and the voltage of the leg can be expressed as:

12 $$\begin{aligned} {I_{total}} = I_{TEG}\,+ f_I\,I_c {\text { and }} {V_{total}}=V_{TEG}\, + f_V\,V_{ce} \end{aligned}$$

where ${f_{I}}$ and ${f_{V}}$ are, respectively, the fractions of the collector current and the voltage between collector and emitter of the transistor, contributing to the output power of the leg. The output power of the new leg is then computed by:

13a $$\begin{aligned} {P_{e}}&= \left( I_{TEG}\,+ f_I\,I_c\right) \,\left( V_{TEG}\,+ f_V\, V_{ce}\right) = \left( I_{TEG}\,+ f_I\,I_c\right) \,V_{TEG} + f_V\,\left( I_{TEG}\, + f_I\,I_c\right) \,V_{ce} \end{aligned}$$

Introducing Eq. (7) into Eq. (13a), the output power can be expressed as:

13b $$\begin{aligned} P_e&= I_{TEG}^2\,R_L\,\left( 1\,+\frac{f_I\,I_c}{I_{TEG}}\right) ^2\, + f_V\,\left( I_{TEG}\,+ f_I\,I_c\right) \left[ V_{cc}\,- \beta \, \frac{R_c}{R_b}\,\left( V_{in} - V_{be}\right) \right] \nonumber \\&= I_{TEG}^2\,R_L\,\left[ 1\,+\frac{f_I\,I_c}{I_{TEG}}\right] ^2\, + f_V\,\left( I_{TEG}\,+ f_I\,I_c\right) \left[ V_{cc}\,-\beta \,\frac{R_c}{R_b}\, V_{in}\right] + f_V\,\left( I_{TEG} + f_I\,I_c\right) \beta \, \frac{R_c}{R_b}\,V_{be}. \end{aligned}$$

Since both the second and the third terms of Eq. (13b) are larger than 0, these two terms can be represented by $\delta P (> 0)$. Equation (13b) can then be approximated by:

13c $$\begin{aligned}&P_e = I_{TEG}^2\,R_L\,\left( 1\,+\frac{f_I\,I_c}{I_{TEG}}\right) ^2\,+ \delta P = P_0\,\left( 1\,+\frac{f_I\,I_c}{I_{TEG}}\right) ^2\,+ \delta P \ge \alpha _P\,P_0. \end{aligned}$$

where ${\alpha _P}$ is denoted as the power gain of the leg.

The improvement in the output power can be measured by the power gain rate defined as:

14 $$\begin{aligned} {\eta _P} = \frac{P_e\,- P_0}{P_0} \approx \left( 1\, +\frac{f_I\,I_c}{I_{TEG}}\right) ^2\,- 1 = \frac{2\,f_I\,I_c}{I_{TEG}} + \left( \frac{f_I\,I_c}{I_{TEG}}\right) ^2 \end{aligned}$$

For a single thermocouple, $I_{TEG}$ is usually smaller than $I_c$. If the term $f_I\,I_c$ in Eq. (14) can be designed to be greater than $I_{TEG}$, the power gain rate can be greater than 3. If the heat rate used to generate $P_0$ and $P_e$ are the same, the efficient gain rate would be the same as $\eta _P$.

The ratio between the output power and the supplied power, i.e.,

15 $$\begin{aligned} {\eta _0} = \frac{P_e}{V_{cc}\,(1-f_I)\,I_c} \end{aligned}$$

can be considered as the effectiveness of the leg for power generation. The effectiveness depends on the fractions of the collector current and the collector-emitter voltage contributed to the output power of the leg. It is worth to note that $I_c$ is the total collector current injected by the emitter. If the transistor is designed such that large portion of the injected carriers enters the thermoelectric loop, the effectiveness of the leg would be large. In addition, the effectiveness is only relevant for the design using a bipolar transistor. When charge carriers are injected through high-doping semiconductors joined with the leg at the leg’s exterior boundary, the power supply is not needed.

## Discussion

A simple example is given here to illustrate the potential of using external carrier injection in improving the efficiency of a thermoelectric leg. The carriers are injected using a bipolar junction transistor as shown in Fig. 4. The uni-leg thermocouple is made of *n*-type Silicon semiconductor and has a cross-sectional area ($A_n$) of 0.267 cm$^2$, a length ($L_n$) of 2 mm, and an initial doping concentration ($N_D$) of 10$^{15}$cm$^{-3}$. The temperature is 300 K at the cold end and 500 K at the hot end. The bipolar transistor is embedded on the exterior surface of the leg. A schematics of the transistor is shown in Fig. 5. The parameters used for each region of the *n*-*p*-*n* transistor are given in Table 2. These parameters are estimated based on those used in Ref^94,95^. The cross-sectional area ($A_t$) of the transistor is 5 $\mu$m$^2$. The supplied voltage $V_{cc}$ is fixed at 3.5 *V*. Three load resistance ($R_c$), 3 $\Omega$, 1.95 $\Omega$, and 1.35 $\Omega$ are used for the collector-emitter loop. The load resistance ($R_b$) for the emitter-base loop is 1 k$\Omega$. The electric current flowing through the thermoelectric loop, including the leg and a load resistance $R_L$, is 0.09 *A* calculated using a thermoelectric model. The calculated mean electrical conductivity of the leg is 32 S/m. The mean intrinsic resistance of the leg is 2.34 $\Omega$ estimated by $R_I=L_n/(\sigma \,A_n)$. The load resistance is $R_L=3.12\,\Omega$ calculated by $10^2/\sigma$ to approximately match the leg intrinsic resistance.

**Table 2:** Parameters used for each region of the *n*-*p*-*n* transistor. Note: (1) the collector is part of the leg body so as to have the same doping density as the thermoelectric leg. (2) the parameter symbol is in the parenthesis following the parameter value.

Region	Doping density	Width	Diffusivity	Lifetime
	cm$^{-3}$	$\mu$m	cm$^2$/s	s
Emitter	$10^{19}\,(N_{De})$	$1\,(x_e)$	$1\,(D_{pe})$	$10^{-8}\,(\tau _{pe})$
Base	$10^{17}(N_{Ab})$	$0.5\,(W)$	$10\,(D_{nb})$	$10^{-7}\,(\tau _{nb})$
Collector	$10^{15}(N_{Dc})$	$3\,(x_c)$	$2\,(D_{pc})$	$10^{-6}\,(\tau _{pc})$

The three electric current equations given by Eqs. (2a), (2b), (2c) and the voltage equations given by Eqs. (6) plus the relation of these voltages given by:

16 $$\begin{aligned}&V_{ce} = V_{cb} + V_{be} \end{aligned}$$

are used to determine the collector current along the load line defined by the second equation of (6). Substituting $V_{ce}$ of Eq. (16) into the second equation of (6), the supplied voltage can be expressed as:

17 $$\begin{aligned}&V_{cc} = R_c\,I_c + V_{cb} + V_{be} \end{aligned}$$

Introducing $V_{be}$ obtained from Eq. (2b) and $I_c$ of Eq. (3) into Eq. (17), the interception point between current-voltage characteristic curve and the load line can be determined for a given base current, $I_b$.

**Figure 6:**
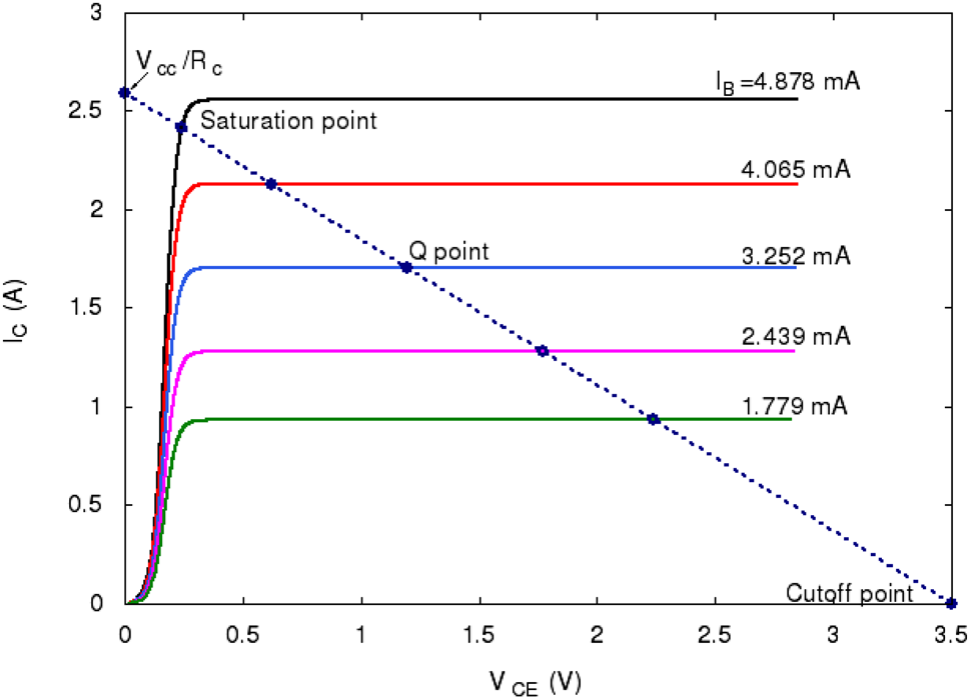
Current-voltage characteristics, load line and their interception points for several specific base currents $I_b$s. The collector doping concentration used to obtain these results is $N_D=2.5\cdot \,10^{15}$cm$^{-3}$. The rest parameters of the transistor are given in the text.

Figure 6 shows the current-voltage curve, the load line, and their interception points for different base currents. The interception point with maximum $I_c$ and $I_b$ is the saturation point below which both the base-emitter and the base-collector junctions are forward biased and the transistor is in saturation mode. The interception point with $I_c=0$ and $V_{ce}=V_{cc}$ is the cutoff point. All the points intercepted by different $I_b$ currents between the saturation and the cutoff points are the quiescent points (Q-point). A transistor always operates at the quiescent points for the given $V_{cc}$ and $R_c$.

Substituting $V_{be}$ of the first equation of (6) into Eq. (17) and using Eq. (3) for $I_b$, the common-emitter collector current along the load line can be determined by:

18 $$\begin{aligned}&I_c = \frac{\left( V_{cc} - V_{cb} - V_{in}\right) + R_b\,\left( a_{12} - \frac{a_{11}\,a_{22}}{a_{21}}\right) \, \left( e^{V_{bc}/V_{th}} - 1\right) }{\left[ R_c\,-\,R_b\,\left( \frac{a_{11}}{a_{21}}\,-\,1\right) \right] } \end{aligned}$$

Setting $V_{be}$ of Eq. (2b) equals to that of the first equation of (6) and in combination with Eq. (18), $V_{bc}$ along the load line can be obtained by solving the following equation:

19 $$V_{{in}} - R_{b} \left[ {\left( {\frac{{a_{{11}} }}{{a_{{21}} }} - 1} \right)I_{c} + \left( {a_{{12}} - \frac{{a_{{11}} a_{{22}} }}{{a_{{21}} }}} \right)\left( {e^{{V_{{bc}} /V_{{th}} }} - 1} \right)} \right] - V_{{th}} \ln \left\{ {1 + \frac{1}{{a_{{21}} }}\left[ {I_{c} - a_{{22}} \left( {e^{{V_{{bc}} /V_{{th}} }} - 1} \right)} \right]} \right\} = 0$$

**Figure 7:**
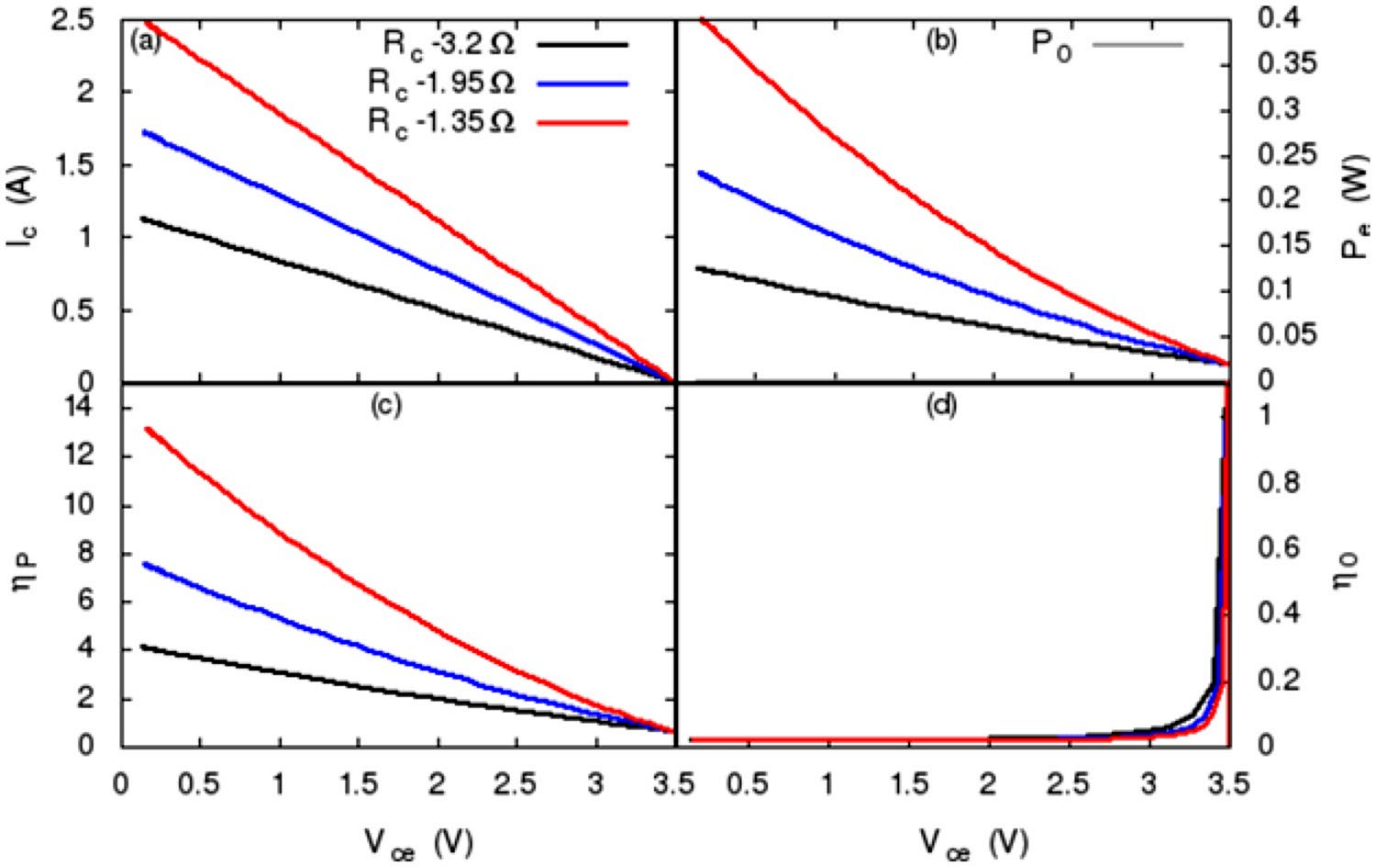
Performance of the new thermoelectric leg. (**a**) Load lines of the amplifier obtained for three $R_c$s. (**b**) Power generated by the thermoelectric leg. $P_0$ power produced solely through Seebeck effect. (**c**) Power gain rate. (**d**) The effectiveness of the new thermoelectric leg. The parameters of the thermoelectric leg and the amplifier used to obtain these results are given in the text.

Figure 7 shows the load lines, the output power, and the power gain rate of the thermoelectric leg obtained using Eqs. (18), (19), (13), (14), and (15) for 3 different $R_c$s: 3.2 $\Omega$. 1.95 $\Omega$, and 1.35 $\Omega$. Figure 7a shows the common-emitter collector currents along the three load lines. As it is known from Fig. 6, the base current, $I_b$, varies along load line and $I_b$ decreases with increase of the collector-emitter voltage, $V_{ce}$. Based on this knowledge, it can be recognized from Fig. 7a that $I_c$ level depends on both the base current $I_b$ and the slope ($1/R_c$) of the load line. Larger $I_b$ and smaller $R_c$ produce a larger $I_c$. Figure 7b shows the power generated by this new thermoelectric leg. The amount of the generated power has a similar trend as the load line but nonlinear, that is, increases nonlinearly with $I_b$ and $1/R_c$. Figure 7b also shows the power generated solely through Seebeck effect, $P_0$, in gray line. Figure 7c shows the power gain rate defined by Eq. (14). As it is showed in Fig. 7a that the magnitude of $I_c$ is significantly larger than the thermoelectric current $I_{TEG}$ due to Seebeck effect, therefore, the power gain rate is significant. The power gain rate also depends on the base current ($I_b$) and the slope of the load line ($1/R_c$). Figure 7d shows the ratio between the generated power and supplied power, i.e., $P_e/[V_{cc}\,(1-f_I)\,I_c]$. As noted earlier, the collector current is significantly larger than the thermoelectric current $I_{TEG}$ of the leg and the fraction that $I_c$ contributed to $P_e$ is small ($f_I=0.1$ is assumed). Consequently, the effectiveness is smaller for most of the $I_c$ on the load line. For smaller $I_c$ near the cutoff point of the load line, the effectiveness can be larger than 1 but the power gain rate is low. Evidently, there exists a trade off between the power gain ratio and the effectiveness when designing such a thermocouple. It worth to emphases that when high doping semiconductor is used as the carrier injector rather than the BJTs, the effectiveness parameter is irrelevant.

## Conclusions

From the relations between thermoelectric properties (Seebeck coefficient, electrical conductivity, thermal conductivity, power factor, and figure of merit) and doping concentration, an optimal figure of merit can be attainable by rigidly shifting the electrical conductivity curve toward low doping concentration without disturbing the Seebeck coefficient and the thermal conductivity curves. This is in contrast to the current high-doping concentration approach in finding an optimal figure of merit among various class of materials.

The proposed new thermocouple design that uses low doping Silicon as the thermoelectric legs and bipolar junction transistors as the external carrier injectors can triple the power output and efficiency of the original design when the electric current in the leg contributed by the injected carriers is equal to or greater than the current induced by the Seebeck effect. The improvement on the power output and the efficiency of the new thermocouple significantly depends on the base current and the load line slope of the transistor.

This work presents a typical example to improve the figure of merit of a thermocouple through its design such that its efficiency and power output can potentially exceed those of the materials of the thermoelectric legs. Using low-doping material as thermoelectric leg and high-doping semiconductors as external carrier injectors is a new concept for thermocouple design and can potentially lead to the development of new thermoelectric materials and high-efficient thermoelectric modules.

## Methods

### Determining current-voltage characteristics of BJT

Under normal operating condition, i.e., the forward active mode, the emitter-base junction is forward biased, whereas the collector-based junction is reverse biased. In this mode, the electron concentration in the base at the emitter-base junction is much higher than the equilibrium concentration of minority carriers in the base. Based on ideal *p*-*n* junction theory, the electron concentration in the base at the emitter-base junction can be expressed as:

20 $$\begin{aligned} {n_{be}} = n_b\left( x_e\right) = n_{b0}\,e^{{V_{be}}/{V_{th}}} \gg n_{b0} \end{aligned}$$

where ${n_{b}}$ is the electron concentration in the base, ${x_{e}}$ the boundary of the depletion region of the emitter-base junction at the emitter side, ${V_{th}}=k_B\,T/q$ is the thermal voltage, $V_{be}$ is the voltage drop across the junction ($V_{be} \gg V_{th}$). The equilibrium concentration of minority carriers in the base, ${n_{b0}}$, is calculated by $n_{b0}=n_{ib}^2/N_{Ab}$, where ${n_{ib}}$ and $N_{Ab}$ are, respectively, the intrinsic carrier and the acceptor concentrations of the base.

To the contrary, the electron concentration in the base at the collector-base junction is much smaller than the equilibrium concentration of minority carriers in the base and is given by:

21 $$\begin{aligned} {n_{bc}} = n_b\left( x_e + W\right) = n_{b0}\,e^{{V_{bc}}/{V_{th}}} \ll n_{b0} \end{aligned}$$

where $V_{bc}$ is the collector-base voltage ($V_{bc} < 0$ for forward active mode), and *W* is the base width. Here, it is assumed that the widths of the depletion regions at the emitter-base and collector-base interface are sufficiently small and can be neglected comparing to the base width.

The distribution of minority carrier in the base region can be determined by the one-dimensional steady-state continuity equation:

22 $$\begin{aligned}&D_{nb}\,\frac{d^2 n_b(x)}{dx^2} = \frac{n_b(x) - n_{b0}}{\tau _{nb}}\, \quad \,\Longrightarrow \,\quad \, \frac{d^2 \Delta n_b(x)}{dx^2} = \frac{\Delta n_b(x)}{L_{nb}^2} \end{aligned}$$

in which ${\Delta n_{b}}(x)=n_b(x)-n_{b0}$ and ${L_{nb}}=\sqrt{D_{nb}\,\tau _{nb}}$ is the diffusion length, where ${D_{nb}}$ and ${\tau _{nb}}$ are, respectively, the diffusivity and the lifetime of electron in the base.

The solution of the continuity equation, Eq. (22), is straightforward. For the two boundaries given by Eqs. (20) and (21), the solution can be written as:

23 $$\begin{aligned} \Delta n_b(x)&= \left[ \Delta n_b(x_e + W)\, \frac{\sinh {\left( \frac{x-x_e}{L_{nb}}\right) }}{\sinh {\left( W/L_{nb} \right) }} - \Delta n_b(x_e)\, \frac{\sinh {\left( \frac{x-x_e-W}{L_{nb}}\right) }}{\sinh {\left( W/L_{nb} \right) }}\right] . \end{aligned}$$

Using a one-dimensional continuity equation identical to Eq. (22) and using the minority carrier concentrations at the boundaries of the emitter: $p_e(0)={p_{e0}}$ and $p_e(x_e)=p_{e0}\,e^{V_{be}/V_{th}}$, the distribution of hole concentration in the emitter, ${p_{e}}(x)$, can be determined by:

24 $$\begin{aligned} \Delta p_e(x) = \frac{p_{e0}}{\sinh {\left( x_e/L_{pe}\right) }} \left( e^{V_{be}/V_{th}} -1\right) \,\sinh {\left( \frac{x}{L_{pe}}\right) }. \end{aligned}$$

where ${\Delta p_{e}}(x)=p_e(x)-p_{e0}$, ${L_{pe}}=\sqrt{D_{pe}\,\tau _{pe}}$ is the hole diffusion length, in which ${D_{pe}}$ and $\tau _{pe}$ are, respectively, the diffusivity and lifetime of hole in the emitter, $p_{e0}={p_{ie}}^2/N_{De}$, $p_{ie}$ the intrinsic hole concentration, and $N_{De}$ the donor concentration in the emitter.

Similarly, using the minority carrier concentrations at the boundaries of the collector: $p_c(x_e+W)=p_{c0}\,e^{V_{bc}/V_{th}}$ and ${p_{c}}(x_e+W+{x_c})={p_{c0}}$, the hole concentration in the collector, $p_c(x)$, can be found and is given by:

25 $$\begin{aligned} \Delta p_c(x) = \frac{p_{c0}}{\sinh {\left( x_e/L_{pc}\right) }} \left( e^{V_{bc}/V_{th}} -1\right) \,\sinh {\left( \frac{x_e+W+x_c-x}{L_{pc}}\right) } \end{aligned}$$

where ${\Delta p_{c}}(x)=p_c(x)-p_{c0}$, ${L_{pc}}=\sqrt{D_{pc}\,\tau _{pc}}$ is the hole diffusion length, in which ${D_{pc}}$ and ${\tau _{pc}}$ are, respectively, the diffusivity and lifetime of hole in the collector, $p_{c0}={p_{ic}}^2/N_{Dc}$ the equilibrium hole concentration, and $N_{Dc}$ the donor concentration of the collector.

Electric current induced by carrier diffusion is proportional to the spatial derivative of the carrier concentration. The diffusion current in the base at the emitter edge is thus given by:

26 $$\begin{aligned}&{I_{ne}} = q\,S\,D_{nb}\,\left[ \frac{\partial n_b(x)}{\partial x}\right] _{x=x_e} = \frac{q\,D_{nb}\,S}{L_{nb}} \left[ \frac{\Delta n_b(x_e + W)}{\sinh {\left( W/L_{nb} \right) }} - \Delta n_b(x_e)\,\coth {\left( \frac{W}{L_{nb}}\right) }\right] \end{aligned}$$

where *S* is the cross-sectional area of the base region.

The diffusion current in the base at the collector edge is determined by:

27 $$\begin{aligned}&{I_{nc}} = q\,D_{nb}\,S\,\left[ \frac{\partial n_b(x)}{\partial x} \right] _{x=x_e+W} = \frac{q\,D_{nb}\,S}{L_{nb}} \left[ \frac{\Delta n_b(x_e + W)}{\coth {\left( W/L_{nb} \right) }} - \frac{\Delta n_b(x_e)}{\sinh {\left( \frac{W}{L_{nb}}\right) }}\right] \end{aligned}$$

The electric current caused by hole diffusion in the emitter at the emitter-base junction is determined by:

28 $$I_{{pe}} = qD_{{pe}} S \left[ {\frac{{\partial p_{e} (x)}}{{\partial x}}} \right]_{{x = x_{e} }} = \frac{{q D_{{pe}} Sp_{{e0}} }}{{L_{{pe}} }}\left( {e^{{\eta _{{be}} }} - 1} \right)\coth \left( {\frac{{x_{e} }}{{L_{{pe}} }}} \right)$$

Similarly, the electric current caused by hole diffusion in the collector at the collector-base junction is calculated by:

29 $$I_{{pc}} = qD_{{pc}} S\left[ {\frac{{\partial p_{c} (x)}}{{\partial x}}} \right]_{{x = x_{e} + W}} = \frac{{ - q D_{{pc}} Sp_{{e0}} }}{{L_{{pc}} }}\left( {e^{{\eta _{{bc}} }} - 1} \right)\coth \left( {\frac{{x_{c} }}{{L_{{pc}} }}} \right)$$

These emitter, base, and collector currents can be obtained and expressed in the following compact form:

30a $$\begin{aligned} I_e&= I_{ne} + I_{pe} = a_{11}\,\left( e^{\eta _{be}} -1\right) + a_{12}\,\left( e^{\eta _{bc}} -1\right) \end{aligned}$$

30b $$\begin{aligned} I_c&= I_{nc} + I_{pc} = a_{21}\,\left( e^{\eta _{be}} -1\right) + a_{22}\,\left( e^{\eta _{bc}} -1\right) \end{aligned}$$

30c $$\begin{aligned} I_e&= I_b + I_c \end{aligned}$$

where

30d $$\begin{aligned}&a_{11} = q\,S\,\left[ \frac{D_{pe}\,p_{e0}}{L_{pe}}\, \coth {\left( \frac{x_e}{L_{pe}}\right) } + \frac{D_{nb}\,n_{b0}}{L_{nb}}\, \coth {\left( \frac{W}{L_{nb}}\right) }\right] \end{aligned}$$

30e $$\begin{aligned}&a_{12} = -\frac{q\,S\,D_{nb}\,n_{b0}}{L_{pe} \sinh {\left( \frac{W}{L_{nb}}\right) }} a_{21}=-a_{12} \end{aligned}$$

30f $$\begin{aligned}&a_{22} = -q\,S\,\left[ \frac{D_{nb}\,n_{b0}}{L_{nb}}\, \coth {\left( \frac{W}{L_{nb}}\right) } + \frac{D_{pc}\,p_{c0}}{L_{pc}}\, \coth {\left( \frac{x_c}{L_{pc}}\right) }\right] \end{aligned}$$

When $I_b$ and $V_{bc}$ are given, the common-emitter collector current can be obtained from Eqs. (30a), (30b), and (30c) as:

31 $$\begin{aligned} I_c = \frac{a_{21}}{a_{11}-a_{21}}\,I_b + \left[ \frac{a_{11}\,a_{22} -a_{12}\,a_{21}}{a_{11}-a_{21}}\right] \left( e^{V_{bc}/V_{th}} -1\right) \end{aligned}$$

## Abbreviations

*α*_*F*_: Current gain of a common-base transistor
*α*: Seebeck coefficient
*α*_*P*_: Power gain of the new thermoelectric module
*α*_*T*_: Base transport factor
*β*: Common-emitter current gain
*η*: Thermal-to-electrical energy conversion efficiency
*η*_0_: Effectiveness of the new thermoelectric module
*η*_*bc*_: Voltage ratio: *qV*_*bc*_/(*k*_*B*_*T*) with *k*_*B*_ being Boltzmann constant
*η*_*be*_: Voltage ratio: *qV*_*bc*_/(*k*_*B*_*T*)
*η*_*P*_: Power gain rate of the new thermoelectric module
*γ*: Emitter injection efficiency
*κ*: Thermal conductivity
*κ*_*n*_: Thermal conductivity of an n-type leg
*κ*_*p*_: Thermal conductivity of a p-type leg
*μ*: Carrier mobility
*ρ*_*n*_: Electrical resistivity of an n-type leg
*ρ*_*p*_: Electrical resistivity of a p-type leg
*σ*: Electrical conductivity
*τ*_*nb*_: Lifetime of electron in the base
*τ*_*pc*_: Lifetime of hole in the collector
*τ*_*pe*_: Lifetime of hole in the emitter

## Glossary of terms

*A*_*n*_: Cross-sectional area of an *n*-type leg.
*A*_*p*_: Cross-sectional area of a *p*-type leg.
*D*_*np*_: Diffusivity of electron in the base.
*D*_*pc*_: Diffusivity of hole in the collector.
*D*_*pe*_: Diffusivity of hole in the emitter.
*I*_*b*_: Base electric current.
*I*_*c*_: Collector electric current.
*I*_*e*_: Emitter electric current.
*I*_*TEG*_: Electric current of a thermoelectric module.
*I*_*nc*_: Electron diffusion current in the base at the base-collector junction.
*I*_*ne*_: Electron diffusion current in the base at the base-emitter junction.
*I*_*pc*_: Hole diffusion current in the collector at the base-collector junction.
*I*_*pe*_: Hole diffusion current in the emitter at the base-emitter junction.
*I*_*total*_: Total electric current of a thermoelectric module.
*I*: Electric current.
*K*_*TEG*_: Thermal conductance of a thermoelectric module.
*K*: Thermal conductance.
*L*_*n*_: Length of an *n*-type leg.
*L*_*p*_: Length of a* p*-type leg.
*L*_*nb*_: Diffusion length of electron in the base.
*L*_*pc*_: Diffusion length of holes in the collector.
*L*_*pe*_: Diffusion length of holes in the emitter.
*PF*: Thermoelectric power factor.
*P*_0_: Output power of the original thermoelectric module.
*P*_*e*_: Output power of the new thermoelectric module.
*Q*_*C*_: Heat flux at cold side of a thermoelectric module.
*Q*_*H*_: Heat flux at hot side of a thermoelectric module.
*R*_*I*_: Intrinsic electrical resistance.
*R*_*L*_: Electrical load resistance.
*R*_*b*_: Base circuit load resistance.
*R*_*c*_: Supply circuit load resistance.
*R*_*nc*_: Circuit load resistance for n-p-n transistor.
*R*_*pc*_: Circuit load resistance for p-n-p transistor.
*R*: Electrical resistance.
*S*: Cross-sectional area of the base region.
*T*_*c*_: Absolute temperature at cold side.
*T*_*h*_: Absolute temperature at hot side.
*V*_*be*_: Base-emitter bias.
*V*_*cc*_: Supplied positive voltage.
*V*_*ce*_: Collector-emitter bias.
*V*_*in*_: Input signal voltage.
*V*_*out*_: Output signal voltage equals *V*_*ce*_.
*V*_*th*_: *k*_*B*_*T*/*q*—thermal voltage.
*V*_*total*_: Total terminal voltage of a thermoelectric module.
*W*: Width of the base.
*ZT*: Dimensionless figure of merit of thermocouple.
Δ*T*: Absolute temperature difference.
Δ*V*_*in*_: Variation of input signal voltage.
Δ*V*_*out*_: Variation of output signal voltage.
Δ*n*_*b*_: Excess electron concentration in the base.
Δ*n*: Incremental carrier concentration.
Δ*p*_*c*_: Excess hole concentration in the collector.
Δ*p*_*e*_: Excess hole concentration in the emitter.
*F*_*I*_: Fraction of the collector current.
*F*_*V*_: Fraction of the collector-emitter bias.
*n*_*e*_: Carrier concentration.
*n*_*b*0_: Equilibrium concentration of minority carrier in the base.
*n*_*bc*_: Electron concentration in the base at the base-collector junction.
*n*_*be*_: Electron concentration in the base at base-emitter junction.
*n*_*b*_: Electron concentration of the base.
*n*_*ib*_: Intrinsic carrier concentration of the base.
*p*_*c*0_: Hole concentration at the far-end of the collector (relative to the base-collector junction).
*p*_*c*_: Hole concentration in the collector.
*p*_*e*0_: Hole concentration at the far-end of the emitter (relative to the base-emitter junction).
*p*_*e*_: Hole concentration in the emitter.
*p*_*ic*_: Intrinsic hole concentration in the collector.
*p*_*ie*_: Intrinsic hole concentration in the emitter.
*q*: Electric charge.
*x*_*c*_: Length of the collector region.
*x*_*e*_: Length of the emitter region.
*z**T*: Dimensionless figure of merit of thermoelectric material.
BJT: Bipolar junction transistor.
FGTEM: Functional graded thermoelectric material.
MOSFET: Metal-oide-semiconductor field efffect transistor.
PGEC: Phonon glass electron crystal.
